# Mitochondrial miR-762 regulates apoptosis and myocardial infarction by impairing ND2

**DOI:** 10.1038/s41419-019-1734-7

**Published:** 2019-06-24

**Authors:** Kaowen Yan, Tao An, Mei Zhai, Yan Huang, Qi Wang, Yunhong Wang, Rongcheng Zhang, Tao Wang, Jing Liu, Yuhui Zhang, Jian Zhang, Kun Wang

**Affiliations:** 1 Center for Developmental Cardiology, Institute for Translational Medicine, College of Medicine, Qingdao University, 266021 Qingdao, China; 2 State Key Laboratory of Cardiovascular Disease, Heart Failure center, Fuwai Hospital, National Center for Cardiovascular Diseases, Chinese Academy of Medical Sciences, Peking Union Medical College, 100037 Beijing, China

**Keywords:** Apoptosis, miRNAs, Translation

## Abstract

Mitochondrial dysfunction plays a major role in the pathogenesis of cardiovascular diseases. MicroRNAs (miRNAs) are small RNAs that act as negative regulators of gene expression, but how miRNAs affect mitochondrial function in the heart is unclear. Using a miRNA microarray assay, we found that miR-762 predominantly translocated in the mitochondria and was significantly upregulated upon anoxia/reoxygenation (A/R) treatment. Knockdown of endogenous miR-762 significantly attenuated the decrease in intracellular ATP levels, the increase in ROS levels, the decrease in mitochondrial complex I enzyme activity and the increase in apoptotic cell death in cardiomyocytes, which was induced by A/R treatment. In addition, knockdown of miR-762 ameliorated myocardial ischemia/reperfusion (I/R) injury in mice. Mechanistically, we showed that enforced expression of miR-762 dramatically decreased the protein levels of endogenous NADH dehydrogenase subunit 2 (ND2) but had no effect on the transcript levels of ND2. The luciferase reporter assay showed that miR-762 bound to the coding sequence of ND2. In addition, knockdown of endogenous ND2 significantly decreased intracellular ATP levels, increased ROS levels, reduced mitochondrial complex I enzyme activity and increased apoptotic cell death in cardiomyocytes, which was induced by A/R treatment. Furthermore, we found that the inhibitory effect of miR-762 downregulation was attenuated by ND2 knockdown. Thus, our findings suggest that miR-762 participates in the regulation of mitochondrial function and cardiomyocyte apoptosis by ND2, a core assembly subunit of mitochondrial complex I. Our results revealed that mitochondrial miR-762, as a new player in mitochondrial dysfunction, may provide a new therapeutic target for myocardial infarction.

## Introduction

Cardiovascular diseases are considered the main causes of human mortality worldwide^1^. Mitochondrial-based disturbances in energy supply, calcium homeostasis, reactive oxygen species generation and cell death have major roles in the pathogenesis of heart failure^2^. Nevertheless, the underlying mechanisms of mitochondrial energy homeostasis are not fully understood.

The human mitochondrial DNA (mtDNA) consists of a circular molecule of 16,569 bp encoding 13 proteins, which is required for oxidative phosphorylation (OXPHOS)^3,4^. The OXPHOS system contains five multiple subunit enzyme complexes, including complexes I, II, III, IV, and V, and one or more of the core subunits for the NADH-ubiquinone oxidoreductase (Complex I) is encoded by mtDNA, such as the mitochondrial NADH dehydrogenase subunit 2 (ND2) protein, which is a core assembly subunit of the mitochondrial respiratory chain complex I and is essential for catalyzing NADH dehydrogenation and electron transfer to ubiquinone to produce ATP^5^. Previous reports showed that mutated ND2 impairs mitochondrial respiratory chain complex I assembly, contributing to Leigh syndrome^6^. The mutant ND2 can increase mitochondrial reactive oxygen species (ROS) production^7^. In addition, ND2 is involved in numerous diseases, including cardiovascular disease, Alzheimer’s disease, and Parkinson’s disease^8^. Japanese individuals with the ND2 mutant have been reported to exhibit a low prevalence of myocardial infarction^9,10^. However, the molecular mechanism of ND2 in myocardial infarction remains to be fully elucidated.

MicroRNAs (miRNAs) are small RNAs that act as negative post-transcriptional gene regulators by inhibiting mRNA translation or promoting mRNA degradation^11,12^. Several reports have revealed that miRNAs play a vital role in mitochondrial ATP production, including miR-15b, miR-16, miR-195, and miR-424^13^. The electron transport chain (ETC) is required for mitochondria to produce ATP via mitochondrial OXPHOS. However, various studies have shown that miRNAs regulate mitochondrial OXPHOS by directly targeting the ETC components in cardiomyocytes. MiR-181c can regulate myocardial mitochondrial energy metabolism by targeting the mRNA of mitochondrial cytochrome c oxidase subunit 1 (COX1)^14^.

Our present work revealed that nuclear miR-762 was translocated into the mitochondria and upregulated in response to A/R treatment. Knockdown of endogenous miR-762 significantly attenuated the decrease in intracellular ATP levels, the increase in ROS levels, the decrease in mitochondrial complex I enzyme activity and the increase in apoptotic cell death in cardiomyocytes, which was induced by A/R treatment. Similarly, knockdown of endogenous miR-762 ameliorated cardiac function in mice. Furthermore, enforced expression of miR-762 dramatically attenuated expression of ND2 by binding to the coding sequence (CDS) of ND2 mRNA. Taken together, our results showed that a novel nuclear miR-762 translocates into the mitochondria and regulates apoptosis and myocardial infarction by impairing ND2 and may serve as a new therapeutic target for myocardial infarction.

## Results

### MiR-762 predominantly translocates into the mitochondria and is upregulated upon A/R treatment

**Fig. 1:**
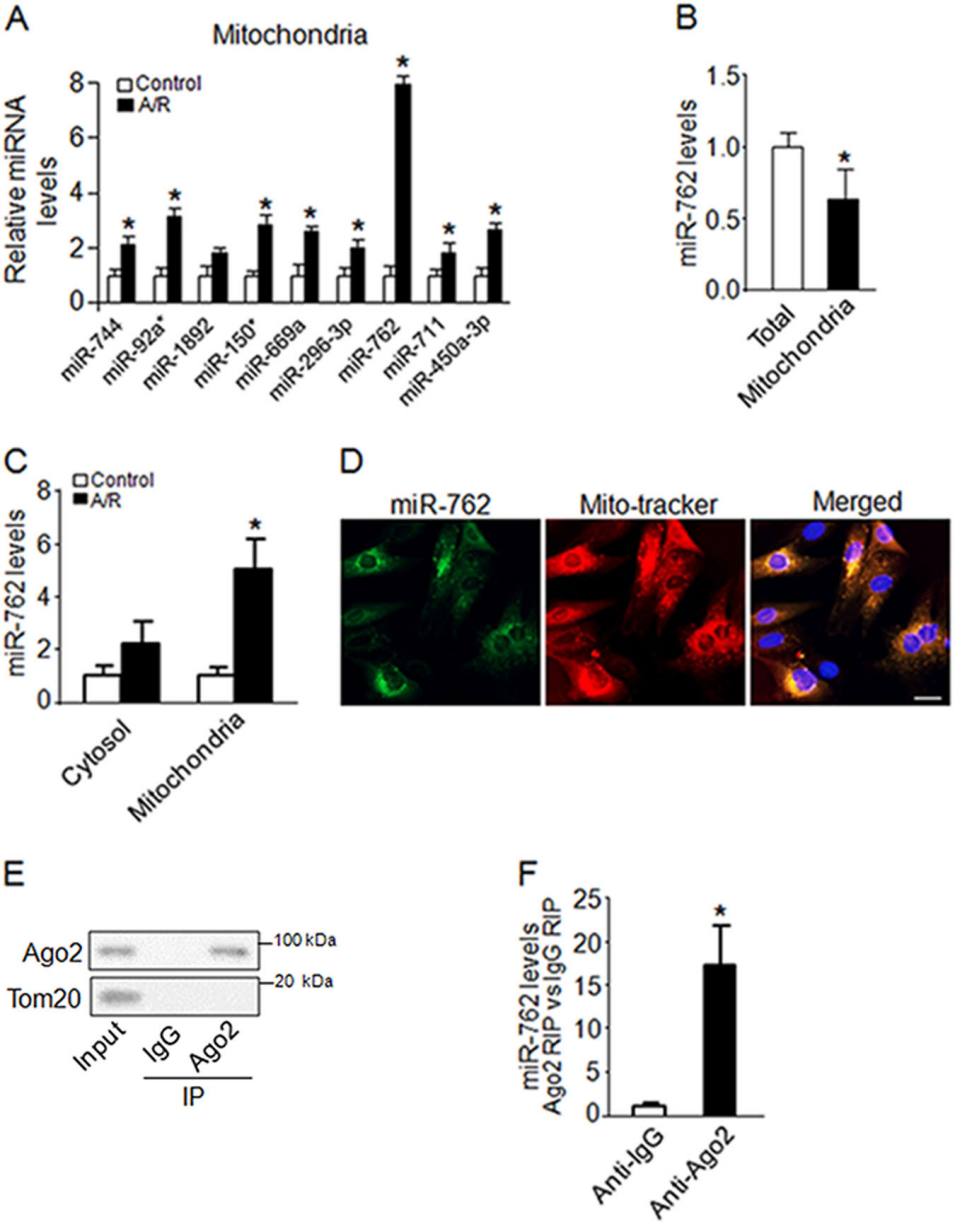
MiR-762 mainly translocates in mitochondria and is upregulated upon A/R treatment. **a** Mitochondrial miRNA expression levels upon A/R treatment. Cardiomyocytes were left untreated (control) or treated with A/R and the expression of myocardial mitochondrial miRNAs in the miRNA array was verified by qRT-PCR (*n* = 3). **b** MiR-762 was predominantly expressed in the cell mitochondria. The miRNA-enriched RNA was extracted from whole cardiomyocytes and myocardial mitochondria fraction, respectively. The levels of miR-762 were analyzed by qRT-PCR (*n* = 3). **c** MiR-762 is dramatically upregulated both in cytosolic and mitochondria upon A/R treatment (*n* = 3). **d** Co-localization of miR-762 with the mitochondrial marker in cardiomyocytes was detected by FISH. MiR-762: green; Mitochondrial marker: Red; Nuclei: blue; Bar = 10 µm. **e**, **f** The translocation of miR-762 into mitochondria was mediated by Ago2. Cell lysates were incubated with non-immune IgG or anti-Ago2 antibody. The Ago2 protein levels and mitochondrial marker protein Tom20 in the input and RIP pull-down materials were detected by western blot analysis as shown in the **e**. The amount of miR-762 in RIP pull-down materials was analyzed by qRT-PCR using miR-762 specific primers in the **f** (*n* = 3). *P < 0.05. Data are shown as the mean ± SEM and analyzed using unpaired student’s *t*-test (**a**–**c**, **f**)

Myocardial apoptosis mediated by mitochondrial dysfunction is an important mechanism for the initiation and progression of heart failure^15^. MiRNAs act as negative regulators of gene expression and play a vital role in the development of heart failure^16^. To explore whether miRNAs participate in the regulation of cardiac mitochondrial function upon A/R treatment, we performed a microarray assay to detect mitochondrial miRNAs in response to A/R treatment (supplementary Table). We identified 15 miRNAs that were differentially expressed in A/R treatment relative to normal cardiomyocytes (fold changes > 2); 9 of these were significantly upregulated, and the other 6 were downregulated (supplementary Table). These data suggest that these 15 miRNAs may be associated with A/R-mediated cardiac mitochondrial function. To confirm the microarray data, we performed quantitative reverse transcription-polymerase chain reaction (qRT-PCR) to verify the mitochondrial miRNA levels. Consistent with the microarray data, miR-762, miR-744, miR-92a, miR-1892, miR-150, miR-669a, miR-296–3p, miR-711, and miR-450a-3p levels were significantly upregulated in cardiomyocytes upon A/R treatment (Fig. 1a). Furthermore, the role of miR-762 in cardiac mitochondria upon A/R treatment remains unclear. Thus, we chose miR-762 to investigate its functions in cardiac mitochondria following A/R treatment. First, we examined miR-762 expression both in total cardiomyocyte and mitochondrial fractions using qRT-PCR and observed that miR-762 was enriched primarily in the cell mitochondria (Fig. 1b). Second, miR-762 was dramatically upregulated both in the cytosolic and mitochondrial fractions using qRT-PCR upon A/R treatment (Fig. 1c). Third, fluorescence in situ hybridization (Fig. 1d) with miR-762 probes showed colocalization of miR-762 with the mitochondrial marker (MitoTracker Red). Das et al. demonstrated that Ago2 plays an important role in translocating miRNAs into the mitochondria^14^. Since miR-762 is located on chromosome 7, to confirm whether translocation of miR-762 into the mitochondria was mediated by Ago2, RNA immunoprecipitation (RIP) experiment with Ago2 was performed in cardiomyocytes (Fig. 1e). As a result, Ago2 immunoprecipitation showed a high abundance of miR-762 compared with normal IgG immunoprecipitation by using qRT-PCR (Fig. 1f). Thus, these findings suggest that miR-762 is present predominantly in the mitochondria and may regulate mitochondrial function in response to A/R-induced injury.

### MiR-762 regulates mitochondrial function and apoptosis

**Fig. 2:**
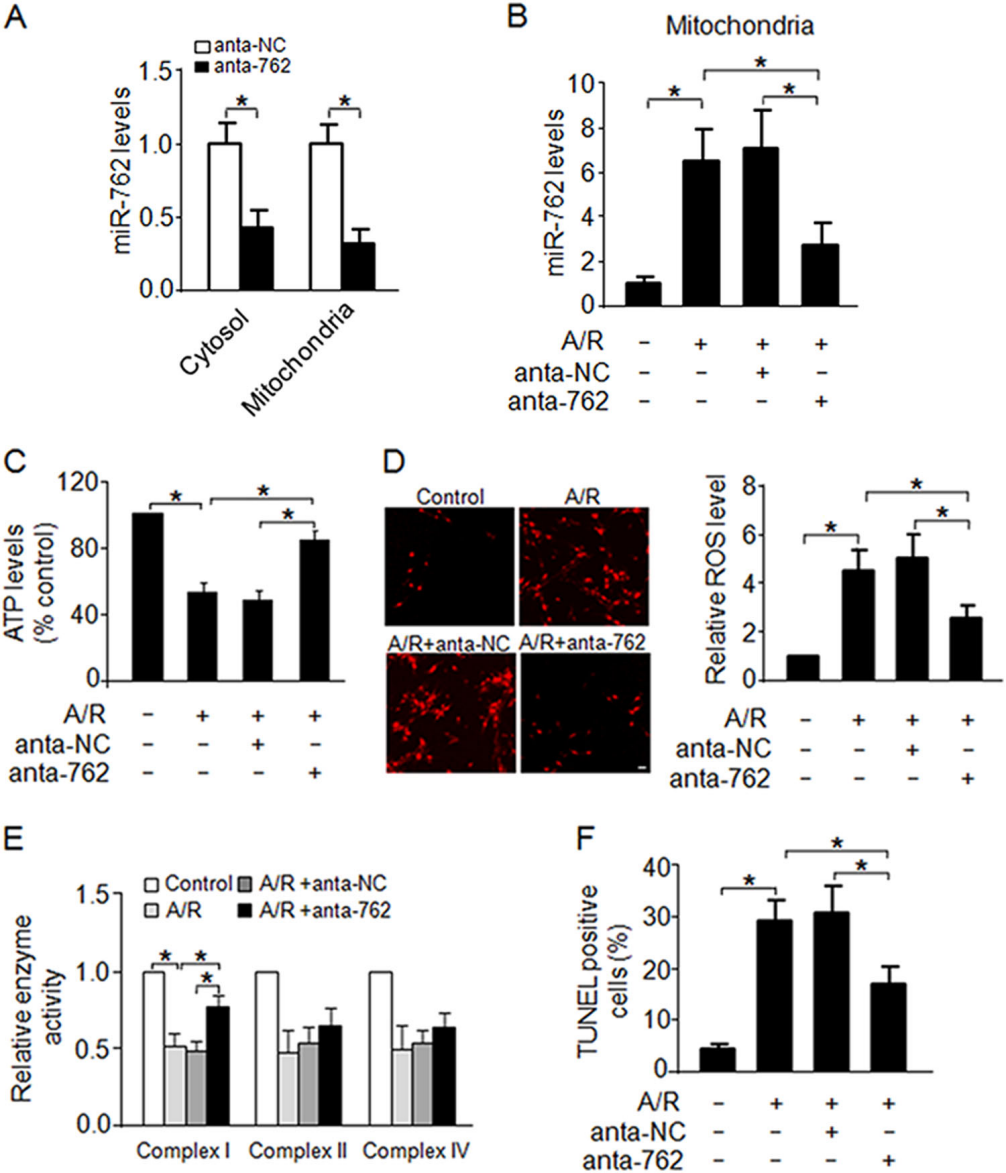
MiR-762 regulates mitochondrial function and apoptosis. **a** Anta-762 dramatically decreased endogenous of miR-762 both in the cytosolic and mitochondria. In vitro cultured cardiomyocytes were transfected with an antagomir for miR-762 (anta-762) or a negative control (anta-NC). The levels of miR-762 in both the cytosolic and mitochondria were detected by qRT-PCR (*n* = 3). **b** Anta-762 significantly attenuated the endogenous levels of miR-762 induced by the A/R treatment. The levels of miR-762 were detected in primary cardiomyocytes which transfected with anta-762 or anta-NC under A/R or no treatment condition, respectively (*n* = 3). **c** Knockdown of miR-762 markedly inhibited the decrease in intracellular ATP levels induced by A/R. Cardiomyocytes were treated as described in **b**, and then, the cellular ATP content was measured with a firefly luciferase-based ATP determination kit (*n* = 3). **d** Knockdown of miR-762 remarkably repressed increase the in ROS levels induced by A/R treatment. Cardiomyocytes were treated as described in **b**, and then, intracellular ROS levels were measured by incubating cardiomyocytes with dichlorodihydrofluorescein diacetate (H2DCFDA, Molecular Probes) (*n* = 3). **e** Knockdown of miR-762 evidently restrained the decrease in enzymatic activity of complex I induced by A/R treatment. Cardiomyocytes were treated as described in **b**, and then, the isolation of an entire mitochondrial fraction from the treated cardiomyocytes was carried out using the Mammalian Mitochondria Isolation Kit. Measurement of mitochondrial complex activities was performed using Microplate assay kits (*n* = 3). **f** Knockdown of miR-762 observably attenuated the increase in apoptotic cell death induced by A/R treatment. Cardiomyocytes were treated as described in **b**, and then, TUNEL staining was used to detect apoptotic cells (*n* = 3). *P < 0.05. Data are shown as the mean ± SEM and analyzed with one-way ANOVA followed by Tukey-Kramer post hoc test (**a**–**f**)

As mentioned above, we observed that A/R treatment induced a significant increase in miR-762 in the mitochondria (Fig. 1a, c). Therefore, we further explored the functional role of miR-762 in the mitochondria. To this end, the antagomir of miR-762 was used to knockdown endogenous miR-762, and we observed that anta-762 dramatically decreased endogenous miR-762 levels both in cardiac cytosol and mitochondria (Fig. 2a). In addition, knockdown of endogenous miR-762 by the antagomir remarkably inhibited the expression of endogenous miR-762 induced by A/R treatment (Fig. 2b). Mitochondria are the center of metabolic signals. In the mitochondrial matrix, tricarboxylic acid circulating enzymes produce electron carriers that form electron transport chains located in the mitochondrial inner membrane to oxygen, which can produce ATP through oxidative phosphorylation^17,18^. MiRNAs were shown to act as regulators of mitochondrial function. For example, miR-15b, miR-16, miR-195 and miR-424 have been shown to regulate ATP levels^13^. Thus, we further explored whether miR-762 also acted as a regulator of ATP levels. Our results revealed that knockdown of miR-762 significantly attenuated the decrease in intracellular ATP levels induced by A/R treatment (Fig. 2c). In addition, our results showed that knockdown of miR-762 attenuated the increase in ROS levels in response to A/R treatment (Fig. 2d). Defects in complexes I-IV, including inefficient electron transfer, produce toxic ROS, which lead to loss of energy metabolism, mitochondrial dysfunction and even human disease^19,20^. MiRNAs regulate mitochondrial complexes; for example, mitochondrial complex III was downregulated by miR-210 in mitochondria; mitochondrial complex IV was downregulated by miR-181c, miR-210, and miR-338^21^. To determine whether the enzyme activity of the mitochondrial complex could be affected by miR-762, we examined the enzyme activities of mitochondrial complexes using a microplate assay kit and found that knockdown of miR-762 apparently repressed the decrease in mitochondrial complex I enzyme activity induced by A/R treatment (Fig. 2e). The oxygen consumption rate (OCR) is an important feature of mitochondrial function, reflecting the mitochondrial respiration rate and energy production. Next, using a Seahorse XFp apparatus, we examined miR-762-mediated responses in cardiomyocytes after miR-762 silencing by anta-762. Our results revealed that A/R treatment induced a significant decrease in basal OCR in cardiomyocytes (supplementary Fig. 1a). And knockdown of miR-762 significantly attenuated the decrease in percentage of ATP-linked OCR in response to A/R treatment (supplementary Fig. 1b). In contrast, knockdown of miR-762 markedly inhibited the increase in percentage of proton leak OCR in response to A/R treatment (supplementary Fig. 1c). Thus, these data further demonstrated that miR-762 exhibits a negative effect on complex I enzyme activity and knockdown of miR-762 may raise efficiency in OXPHOS. Furthermore, knockdown of miR-762 markedly suppressed the increase in A/R-induced apoptosis (Fig. 2f). Overall, these data suggest that miR-762 regulates mitochondrial function and cardiomyocyte apoptosis.

### MiR-762 regulates myocardial infarction and cardiac function

**Fig. 3:**
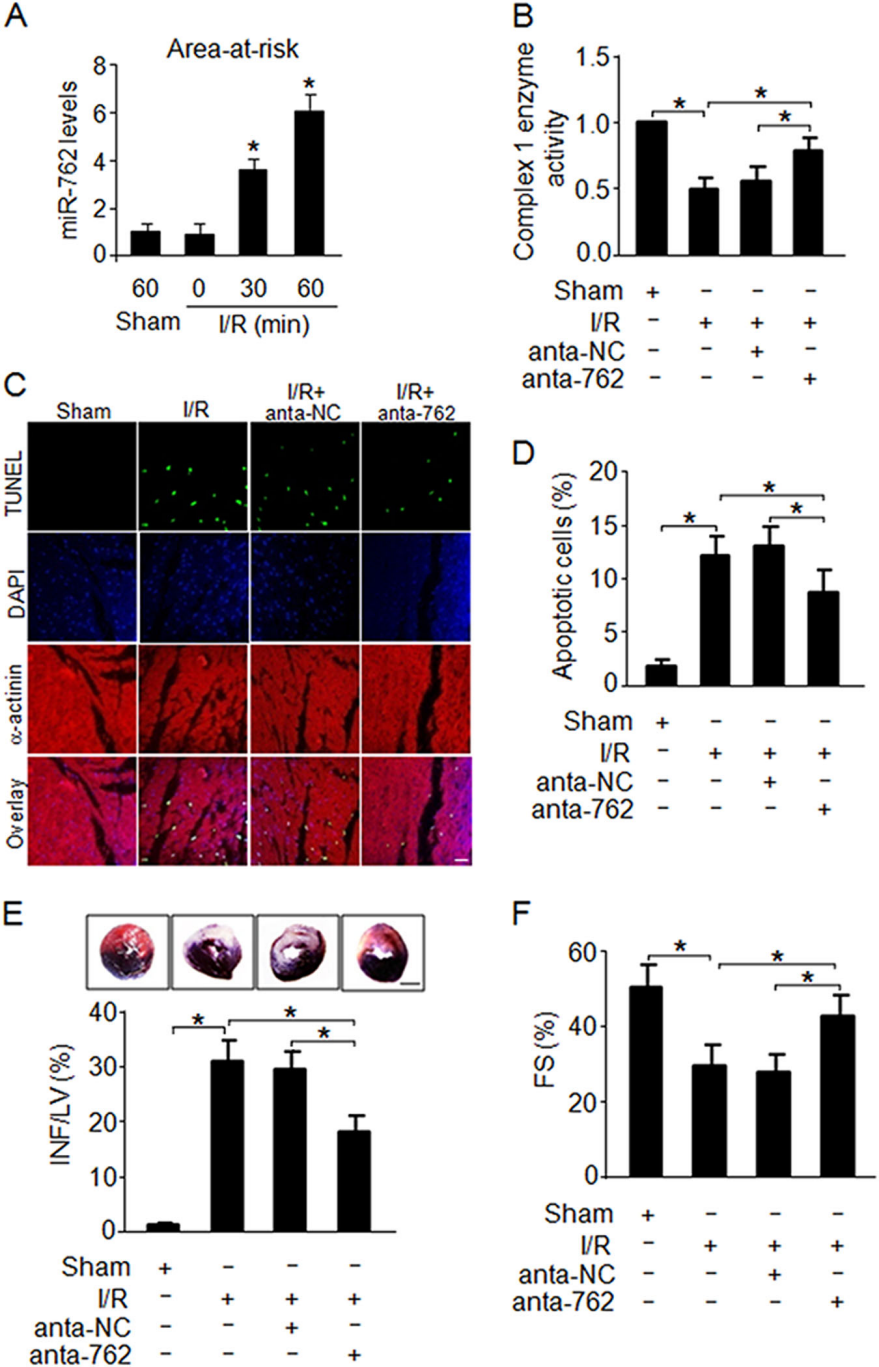
MiR-762 regulates myocardial infarction and cardiac function in the heart. **a** Analysis of miR-762 expression in the area-at-risk under I/R injury. Mice were subjected to I/R surgery at the indicated times and the expression of miR-762 in the area-at-risk was measured by qRT-PCR (*n* = 5). **b** Knockdown of miR-762 by delivery of an antagomir in vivo significantly attenuated the decrease in the enzyme activity of complex I upon I/R injury. Mice were injected with antagomir miR-762 or its control, followed by operating with I/R injury as described in the methods, and then, the enzyme activity of complex I was detected with Microplate assay kits (*n* = 5). **c**, **d** Knockdown of miR-762 by delivery of an antagomir in vivo dramatically inhibited apoptosis induced by I/R injury. Mice were treated as described in **b**, and then, apoptosis was detected by a TUNEL assay. TUNEL-positive cardiomyocyte nuclei (apoptotic cells) are green; nuclei stained by 4’,6-diamidino-2-phenylindole(DAPI) are blue. Cardiomyocytes were labeled with actinin (red) (*n* = 5). Bar = 20 µm. **e**, **f** Knockdown of miR-762 by delivery antagomir in vivo remarkably ameliorated cardiac function in I/R injury. Mice were treated as described in **b**, and then, myocardial infarct size measurement and echocardiographic analysis were performed as described in the Materials and Methods section. FS indicates fractional shortening of left ventricular diameter; LVIDd, diastolic left ventricular internal diameters; and LVIDs, systolic left ventricular internal diameters (*n* = 5). *P < 0.05. Data are shown as the mean ± SEM and analyzed with one-way ANOVA followed by Tukey-Kramer post hoc test (**a**, **b**, **d**–**f**)

Apoptosis plays an important role in post-infarct myocardial remodeling and failure. Preventing myocyte apoptosis contributes to ameliorating cardiac function and cardiac remodeling after myocardial infarction^22^. We showed that miR-762 was involved in regulating A/R-induced myocardial apoptosis (Fig. 2f). Thus, we detected miR-762 expression in the myocardial area at risk in response to I/R injury. As expected, miR-762 was elevated in response to I/R injury (Fig. 3a), suggesting that miR-762 has a potential role in promoting myocardial infarction. To explore the pathophysiological role of miR-762, I/R injury was carried out in mice injected with miR-762 antagomir (anta-762) or its control (anta-NC). Our results showed that knockdown of miR-762 by delivery of the antagomir in vivo remarkably attenuated the decrease in complex I enzyme activity (Fig. 3b) and the increase in apoptotic cells, which was induced by I/R injury (Fig. 3c, d). Likewise, myocardial infarction size in anta-762-treated mice significantly decreased compared with the anta-NC-treated group following I/R injury (Fig. 3e). These results suggest that knockdown of miR-762 contributes to reduced myocyte apoptosis and myocardial infarction. Thus, we further explored whether cardiac function was regulated by miR-762. Our results showed that cardiac function was ameliorated in the anta-762-treated group (Fig. 3f), indicating that knockdown of miR-762 ameliorated myocardial I/R injury in mice. Thus, these findings further support our conclusion that miR-762 regulates myocardial infarction and cardiac function.

### MiR-762 participates in the regulation of ND2 expression

**Fig. 4:**
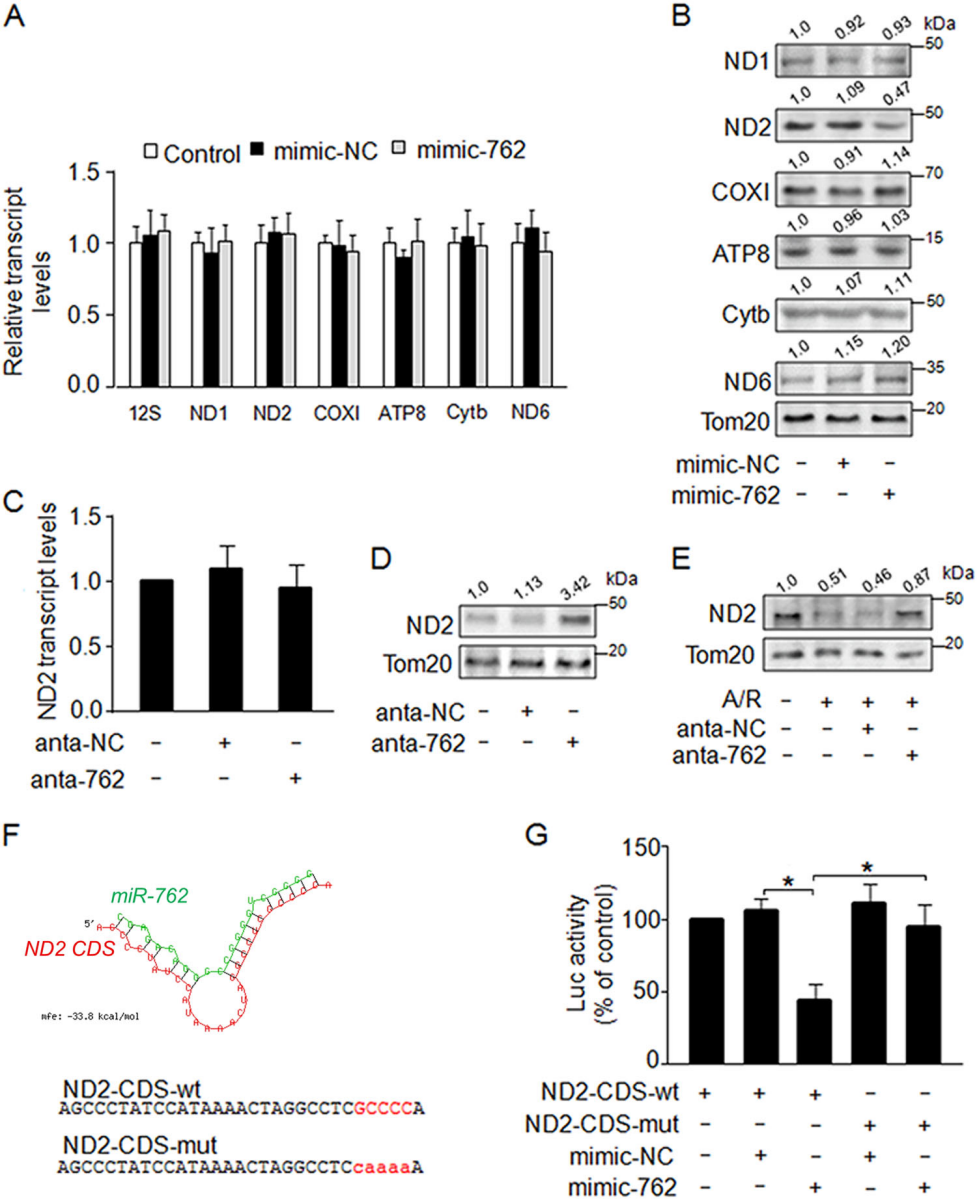
MiR-762 participates in the regulation of ND2 expression. **a** Enforced of miR-762 had no distinct effect on the mRNA levels of mitochondrial protein coding genes. Cardiomyocytes were transfected with mimic-762 or mimic negative control (mimic-NC). The expression of mitochondrial genes was detected by qRT-PCR (*n* = 3). **b** Enforced of miR-762 resulted in a significant reduction in the protein expression of ND2 but had no distinct effect on the other mitochondrial DNA-coded proteins. Cardiomyocytes were treated as described in **a**, and then, the levels of ND2 were analyzed by western blot analysis. **c** Knockdown of miR-762 had no remarkable effect on expression of ND2. Cardiomyocytes were transfected with anta-762 or anta-NC. The mRNA levels of ND2 were analyzed by qRT-PCR (*n* = 3). **d** Knockdown of miR-762 dramatically increased the expression of ND2. Cardiomyocytes were treated as described in (**c**), and then, the expression of ND2 was detected by western blot analysis. **e** Knockdown of endogenous miR-762 attenuated decrease of ND2 induced by A/R treatment. Cardiomyocytes were transfected with an antagomir as described in the methods and followed by A/R treatment. The expression of ND2 was detected by western blot analysis. **f** Analysis of the ND2 CDS binding site for miR-762 and information of mutation sites. **g** MiR-762 significantly inhibited the translation of the luciferase gene by directly binding with the CDS of ND2. HEK293 cells were transfected with a wild-type pGL3-ND2-CDS-WT or mutated pGL3-ND2-CDS-Mut, as well as with a miR-762 mimic or its negative control. The cells were harvested and luciferase activity was measured (*n* = 3). *P < 0.05. Data are shown as the mean ± SEM and analyzed with one-way ANOVA followed by Tukey-Kramer post hoc test (**a**, **c**, **g**)

The mitochondrial genome contains 13 protein-coding genes, two rRNAs and 22 tRNAs^3^. To determine the mitochondrial targets of miR-762, we performed qRT-PCR experiments to investigate whether the transcripts of all 13 mitochondrial protein-coding genes were altered in response to enforced expression of miR-762 by transfection with mimic-762. Our results showed that there was no distinct effect of enforced expression of miR-762 on the transcript levels of 13 mitochondrial protein coding genes (Fig. 4a and supplementary Fig. 2a). Subsequently, we determined the protein levels of all 13 mitochondrial protein coding genes by western blot analysis. We found that compared with the mimic-NC-transfected group, there was a significant reduction in the protein expression of ND2 but no significant effect on other mitochondrial DNA-coded proteins in the mimic-762-transfected group (Fig. 4b and supplementary Fig. 2b). As expected, knockdown of endogenous miR-762 had no striking effect on the transcript levels of endogenous ND2 (Fig. 4c) but effectively increased the protein expression of ND2 (Fig. 4d). Similarly, knockdown of endogenous miR-762 attenuated the reduction in the protein expression of ND2 upon A/R treatment (Fig. 4e). We further carried out the bioinformatic analysis using the RNAhybrid program. Indeed, we noticed that the CDS of ND2 contains a potential binding site for miR-762 (Fig. 4f). Therefore, we investigated the inhibitory mechanism on ND2 translation using a luciferase assay system. We produced a luciferase construct of the wild-type CDS of ND2 (ND2-CDS-wt) and a mutated form (ND2-CDS-mut). The results of the luciferase assay showed that enforced expression of miR-762 significantly inhibited the translation of the luciferase gene containing the wild-type CDS of ND2, indicating that miR-762 directly binds with the wild-type CDS of ND2 (Fig. 4g). Moreover, our results revealed that enforced expression of miR-762 exhibited no distinct effect on the expression of ND2-CDS-mut (supplementary Fig. 3a). In contrast, enforced expression of miR-762 prominently inhibited the expression of ND2-CDS-wt (supplementary Fig. 3b). We further determined intracellular ATP levels, and our results revealed that enforced expression of miR-762 dramatically reduced intracellular ATP levels in the ND2-CDS-wt-transfected group compared with ND2-CDS-mut-transfected group (supplementary Fig. 3c). These findings further support our conclusion that miR-762 directly binds to the CDS of ND2. Collectively, these findings suggest that ND2 is a specific downstream target of miR-762 in the mitochondria.

### ND2 prevents cardiomyocyte apoptosis and ameliorates cardiac function

**Fig. 5:**
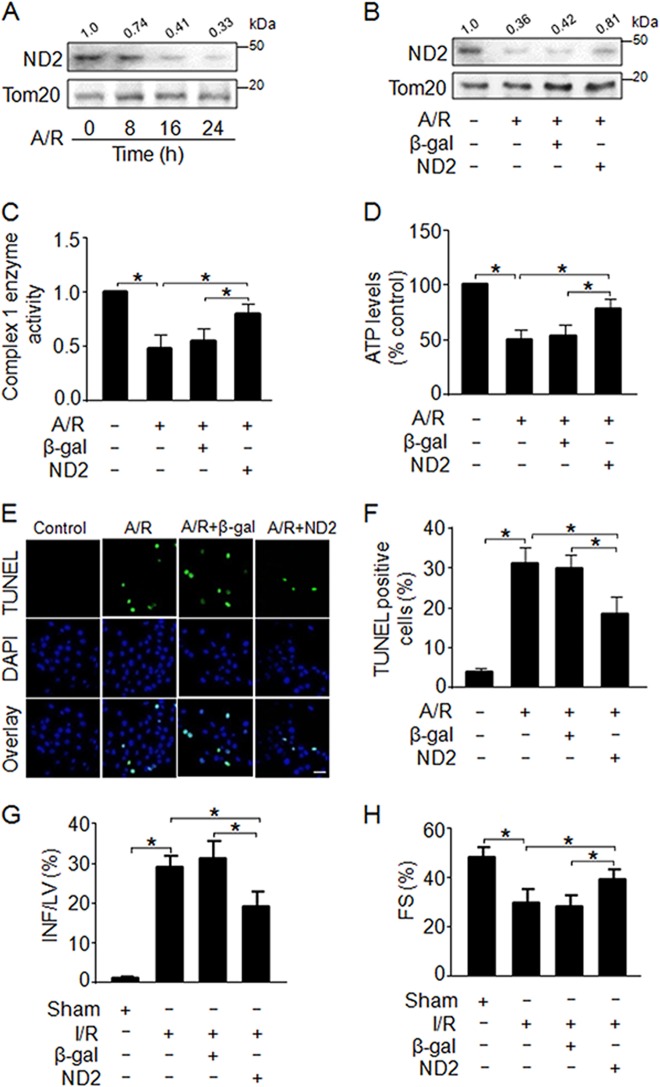
ND2 prevents cardiomyocyte apoptosis by selectively maintaining the activity of mitochondrial complex I. **a** A/R treatment dramatically induced a decrease in ND2 levels. Cardiomyocytes were treated with A/R for the indicated times. ND2 levels were analyzed by western blot analysis. **b** Enforced expression of exogenous ND2 significantly attenuated the reduction in ND2 expression induced by A/R treatment. Cardiomyocytes were infected with adenoviruses harboring ND2 or β-gal. Twenty-four hours after infection, cells were treated with A/R. ND2 levels were analyzed by western blot analyses. **c** Enforced expression of exogenous ND2 evidently inhibited the decrease in the activity of mitochondrial complex I induced by A/R treatment. Cardiomyocytes were treated as described in **b**, and then, the enzyme activity of complex I was detected with Microplate assay kits (*n* = 3). **d** Enforced expression of exogenous ND2 notably repressed the decrease in ATP levels induced by A/R treatment. Cardiomyocytes were treated as described in **b**, and the cellular ATP content was measured with a firefly luciferase-based ATP determination kit (*n* = 3). **e**, **f** Enforced expression of exogenous ND2 prominently restrained the increase in apoptotic cell death induced by A/R treatment. Cardiomyocytes were treated as described in **b** and apoptosis was detected by TUNEL staining (*n* = 3). Bar = 20 µm. **g** Overexpression of ND2 by delivery of adenovirus harboring ND2 in vivo exhibited a significant reduction in myocardial infarction sizes induced by I/R injury. Mice were injected with adenoviruses harboring ND2 or β-gal as described in the methods, and then were subjected to 60 min of 1 week of reperfusion (I/R). INF = infarct size; LV = left ventricular area (*n* = 5). **h** Overexpression of ND2 forcefully ameliorated postischemic myocardial function. Mice were treated as described in **g**. Systolic left ventricular internal diameters (LVIDs) and diastolic left ventricular internal diameters (LVIDd) were measured by echocardiography and Fractional shortening (FS) of the left ventricular diameter was calculated as: FS (%) = [(LVIDd –LVIDs)/LVIDd] × 100 (*n* = 5). *P < 0.05. Data are shown as the mean ± SEM and analyzed with one-way ANOVA followed by Tukey-Kramer post hoc test (**c**, **d**, **f**–**h**)

To explore the role of ND2 in apoptosis under pathological conditions, we observed that A/R dramatically reduced the protein levels of ND2 (Fig. 5a). In contrast, enforced expression of exogenous ND2 significantly attenuated the reduction in ND2 expression induced by A/R treatment (Fig. 5b). Furthermore, we found that enforced expression of ND2 remarkably repressed the decrease in complex I enzyme activity (Fig. 5c) and the reduction in ATP levels, which was induced by A/R treatment (Fig. 5d). In addition, enforced expression of ND2 significantly attenuated A/R-induced apoptotic cell death (Fig. 5e, f). To further explore the function of ND2 in the pathogenesis of myocardial infarction, I/R injury was carried out in the animal model. Our results showed that enforced expression of ND2 by delivery of an adenovirus harboring ND2 in vivo resulted in a significant reduction in myocardial infarction sizes following I/R injury (Fig. 5g). In addition, myocardial function was ameliorated in the mice treated with adenoviral delivery of ND2 in response to I/R injury (Fig. 5h). In general, these data suggest that ND2 plays protective roles against I/R injury-induced apoptosis and ameliorates cardiac function.

### MiR-762 regulates mitochondrial function and apoptosis by ND2

**Fig. 6:**
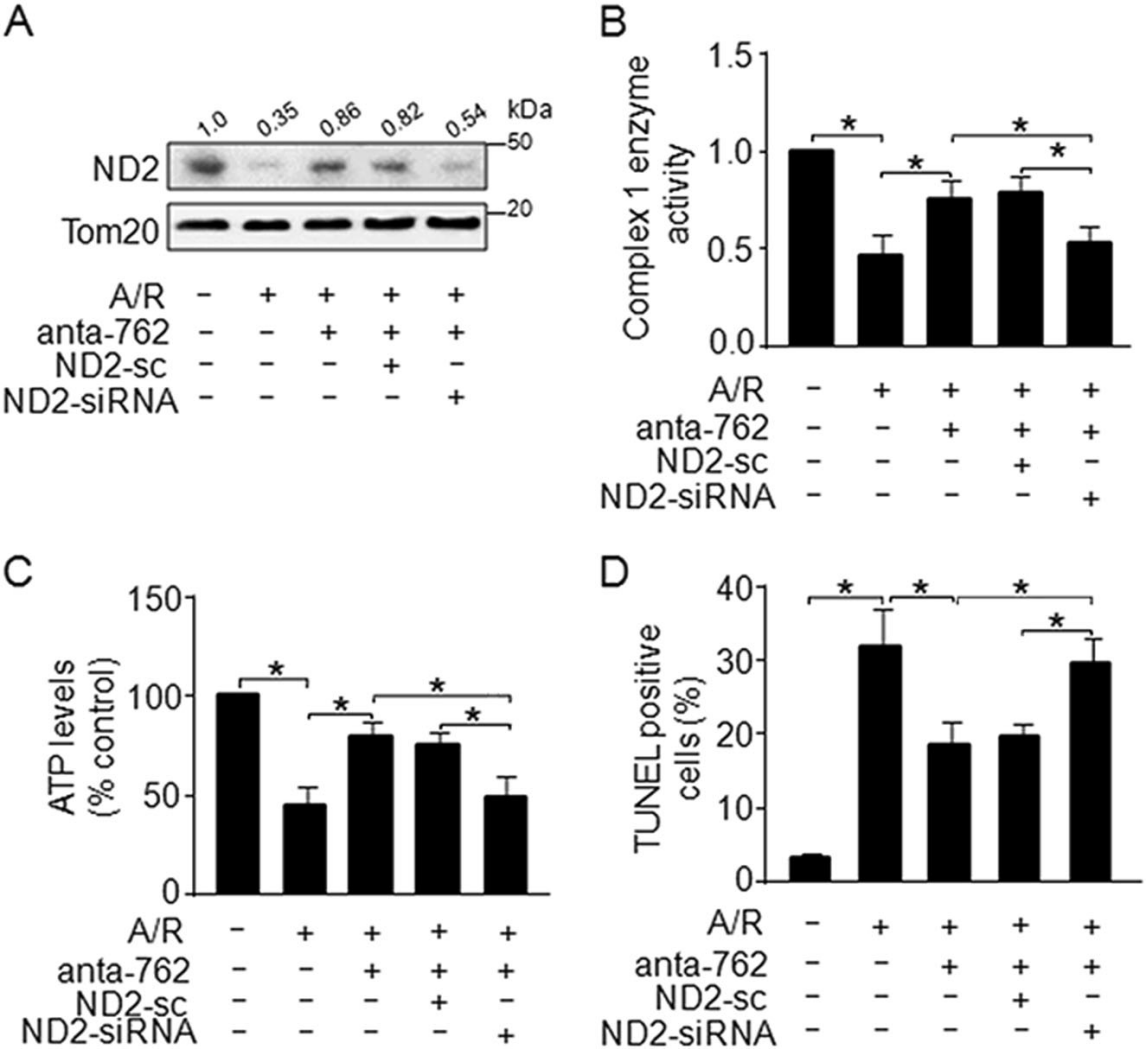
MiR-762 regulates mitochondrial function and apoptosis by ND2. **a** Knockdown of ND2 attenuated the reduction in ND2 induced by miR-762 upon A/R treatment. Cardiomyocytes were transfected with anta-762, ND2-sc or ND2-siRNA with A/R treatment. ND2 levels were analyzed by western blot analysis. **b**, **c** Knockdown of ND2 repressed the protective effect of anta-762 on the enzyme activity of mitochondrial complex I (**b**) and intracellular ATP content (**c**), which was induced by A/R treatment. Cardiomyocytes were treated as described in **a**. The enzyme activity of complex I was detected with Microplate assay kits, and the cellular ATP content was measured with a firefly luciferase-based ATP determination kit (*n* = 3). **d** Knockdown of ND2 attenuated the inhibitory effect of anta-762 on apoptotic cell death in response to A/R treatment. Cardiomyocytes were treated as described in **a** and apoptosis was detected by TUNEL staining (*n* = 3). *P < 0.05. Data are shown as the mean ± SEM and analyzed with one-way ANOVA followed by Tukey-Kramer post hoc test (**b**–**d**)

Knockdown of miR-762 significantly attenuated the decrease in ND2 induced by A/R treatment (Fig. 4e). Thus, we further explored whether miR-762 regulates mitochondrial function and apoptosis by ND2. Our results showed that the inhibitory effect of miR-762 downregulation on ND2 protein levels was attenuated by ND2 knockdown (Fig. 6a). As expected, knockdown of miR-762 significantly repressed the decrease in mitochondrial complex I enzyme activity and intracellular ATP levels, which were induced by A/R treatment (Fig. 2c, e and Fig. 6b, c), and the inhibitory effect of miR-762 downregulation was attenuated by ND2 knockdown (Fig. 6b, c). Our results also showed that knockdown of miR-762 significantly inhibited the increase in apoptotic cell death induced by A/R treatment (Fig. 2f and Fig. 6d),and the inhibitory effect of miR-762 downregulation was attenuated by ND2 knockdown (Fig. 6d). Taken together, these data suggest that miR-762 regulates apoptosis and mitochondrial function by ND2.

## Discussion

MiR-762 is reportedly upregulated in human corneal epithelial cells induced by tear fluid and *Pseudomonas aeruginosa* antigens, which act by targeting a number of important genes, such as RNase7 and ST2^23^. Moreover, miR-762 is involved in bone mineralization by repressing the expression of IFITM5 in Saos-2 cells^24^. In addition, miR-762 was shown to increase breast cancer cell proliferation and invasion by targeting IRF7 expression^25^. Previous research has also demonstrated that miR-762 is upregulated in radiation-induced tumors in mice and is abundant in both breast cancer cell lines and clinical specimens^25,26^. A study demonstrated that miR-762 could inhibit the proliferation of ovarian cancer cells by downregulating the expression of menin through a binding site in the 3’-UTR of menin^27^. However, the role of miR-762 in the heart remains unclear. In our study, we revealed that nuclear miR-762 translocates to the mitochondria and is upregulated in response to A/R treatment. MiR-762 can regulate mitochondrial functions, including inhibition of ATP production and the enzyme activity of complex I, induction of ROS generation and apoptotic cell death. Knockdown of miR-762 ameliorated myocardial I/R injury in mice. Our results suggest that miR-762 is a novel pro-apoptotic miRNA in response to I/R injury in the heart.

To explore the molecular mechanisms by which miR-762 promotes apoptosis and myocardial infarction, ND2 was identified as a specific downstream target of miR-762 in the mitochondria. First, miR-762 reduced ND2 protein levels but had no significant effect on ND2 mRNA in the cardiomyocytes. Second, the CDS of ND2 contains a potential binding site for miR-762. Third, enforced expression of miR-762 significantly inhibited the translation of the luciferase gene containing the CDS of ND2. Fourth, enforced expression of miR-762 dramatically reduced intracellular ATP levels in the ND2-CDS-wt-transfected group compared with the ND2-CDS-mut-transfected group. These results suggest that miR-762, by impairing ND2, promotes myocardial infarction and apoptosis in response to A/R treatment in cardiomyocyte.

In our microarray assay, we identified 15 miRNAs that were differentially expressed in A/R treatment relative to normal cardiomyocytes (fold changes > 2); 9 of these were significantly upregulated, and the other 6 were downregulated (supplementary Table). Consistent with the microarray data, miR-762, miR-744, miR-92a, miR-1892, miR-150, miR-669a, miR-296–3p, miR-711, and miR-450a-3 levels were significantly upregulated in cardiomyocytes upon A/R treatment in our qPCR analysis (Fig. 1a). Mitochondrial miRNAs are generated in the nucleus by RNA polymerase II and further cleaved into pre-miRNA by Drosha. Subsequently, pre-miRNA is transported out of the nucleus to the cytoplasmic exportin 5 and further cleaved into mature miRNA by dicer^28^. Mammals have four argonaute (Ago1–4) proteins, which serve as the main components of the RNA-induced silencing complex (RISC)^29^. In addition, argonaute proteins play important roles in miRNA processing, stability and function^30^. Ago2, the only catalytically active Ago protein, was shown to have multiple roles in small RNA-guided gene silencing processes, including RNA interference, translation repression and heterochromatinization^31^. Furthermore, Ago2 was shown to be enriched in the mitochondria^32^. Studies have shown that Ago acts as a carrier protein that shuttles miRNAs into the mitochondria by binding to the pre-RISC or mature-RISC conformation in the cytoplasm^28^. A previous study showed that miR-1 was translocated into the mitochondria by Ago2 and regulated the translation of mitochondrial ND1 and COX1 proteins during muscle differentiation^33^. In our study, we showed that miR-762 interacts with Ago2 in cardiomyocytes (Fig. 1e). We observed that knockdown of endogenous miR-762 had no significant effect on the transcript levels of ND2 (Fig. 4c) but effectively increased the protein expression of ND2 (Fig. 4d). We also revealed that Ago2 immunoprecipitation had high levels of miR-762 compared with normal IgG immunoprecipitation (Fig. 1f), indicating that miR-762 suppressed the expression of ND2 via translational repression of the Ago2 functional mechanism. However, sensors that detected signaling in response to A/R treatment may exist, which induced some miRNAs to translocate into the mitochondria, such as miR-762, miR-744, miR-92a, miR-1892, miR-150, miR-669a, miR-296–3p, miR-711, and miR-450a-3p (supplementary Table) or prevented some miRNAs from translocating into the mitochondria, such as miR-362-5p, miR-532-5p, miR-31, miR-139-5p, miR-330, and miR-379 (supplementary Table). Thus, the mechanisms underlying the increase or decrease of miRNAs induced by A/R treatment warrant further investigation.

In addition to miR-762, miR-744 regulates gastric cancer cell apoptosis through targeting B cell lymphoma-2^34^. The traditional Chinese medicine astragaloside IV was proven to protect rat cardiomyocytes against hypoxia-induced injury via downregulation of miR-23a and miR-92a^35^. The expression of miR-150 was also reported to be dramatically upregulated in the myocardium remote zone, and the ZFAS1-miR-150 axis plays an important role in acute myocardial infarction-induced cardiomyocytes apoptosis^36^. MiR-711 regulated the apoptosis of H9c2 cardiomyocytes in myocardial ischemia reperfusion through mediating the NF-κB signaling pathway^37^. In addition to miRNAs upregulated in response to A/R treatment, downregulated miRNAs were observed in the miRNA array, such as miR-362-5p, miR-532-5p, miR-31, miR-139-5p, miR-330, and miR-379 (supplementary Table). Therefore, the miR-762-ND2 axis is an important mechanism regulating myocardial infarction and apoptosis in cardiomyocytes, but it is not the only one. We speculate that other miRNAs may have roles and mechanisms in regulating myocardial infarction and apoptosis in the heart, and we will study them further in future studies.

Mitochondria are the major ATP production organelles and consist of 5 respiratory chain complexes, including complexes I, II, III, IV and V. The human mitochondrial genome comprises a circular molecule of 16569 bp encoding 13 proteins, and only the subunits of mitochondrial complexes I, III and IV have been reported to be encoded by the mitochondrial genome. Respiratory chain complex I is the largest multi-protein enzyme complex of the mitochondrial electron transfer chain^38^. Studies have shown that ND2 plays an important role in the proton pumping mechanism of eukaryotic complex I, the *ND2*^del1^ mutation results in uncoupling of proton pumping from electron transfer, decreasing OXPHOS efficiency (ADP/oxygen) with normal basal complex-I mediated electron flux^39^. Moreover, studies have shown that mitochondrial mutations in ND2 may account for increased levels of ROS production^40^. Studies also have shown that hypoxia and reoxygenation treatment induced mitochondrial permeability transition pore opening and ROS generation, resulting in a significant decrease in OCR of mitochondria in H9c2 cells^41^. Furthermore, percentage of proton leak OCR is elevated during A/R treatment^42–44^. Similarly, we also observed that A/R treatment induced a significant decrease in basal OCR in cardiomyocytes (supplementary Fig. 1a). Although there were no significant differences in basal OCR between anta-NC-transfected and anta-762-transfected group, knockdown of miR-762 significantly attenuated the decrease in percentage of ATP-linked OCR and the increase in percentage of proton leak OCR in response to A/R treatment (supplementary Fig. 1b, c). Thus, we speculate that A/R treatment may have other mechanisms in regulating mitochondrial respiration and we will study them further in future studies. Knockdown of miR-762 could improve OXPHOS efficiency (ADP/oxygen), thereby generating more ATP and decreasing ROS production to protect cardiomyocytes against A/R-induced injury. In support of this conclusion, knockdown of miR-762 significantly attenuated the decrease in intracellular ATP levels and the increase in ROS levels induced by A/R treatment (Fig. 2c, d). Collectively, our findings suggest that miR-762 exhibits a negative effect on complex I enzyme activity and knockdown of miR-762 may raise OXPHOS efficiency in cardiac mitochondria.

Over the past few years, some nuclear miRNAs have been reported to regulate the mitochondrial proteome, such as miR-338^45^, miR-210^46^, and miR-1^33^. A recent study showed that nuclear miR-181-c inhibits the translation of COX1, which results in oxygen metabolism defects and promotes ROS production^47^. Our present work identified a novel mitochondrial function axis mediated through translocating nuclear miR-762 into mitochondria and binding the CDS of mitochondrial ND2, which could contribute to inhibiting ATP production and inducing ROS generation and apoptotic cell death. Recently, miR-17/92 clusters were reported to cause mitochondrial DNA damage by increasing ROS levels in lung cancers^48^. MiR-128a can also induce ROS production and further inhibits the growth of medulloblastoma cells^49^. In future studies, we will examine whether miR-762 can cause mitochondrial DNA damage by increasing ROS levels. In conclusion, our present work reveals for the first time that the miR-762-ND2 axis mediates apoptosis and myocardial infarction. Our data may have critical therapeutic guidelines for the employment of ND2 in the treatment of mitochondrial function defect-related cardiac diseases.

## Materials and Methods

### Cell culture and A/R treatment

Neonatal mouse cardiomyocytes were isolated from 1-day-old to 2-day-old male mice, which were purchased from the Institute of Laboratory, Animal Science of Chinese Academy of Medical Sciences (Beijing, China). A/R treatment was carried out as previously described^50^. Briefly, the isolated cardiomyocytes were cultured in an anoxic chamber filled with 5% CO_2_ and 95% N_2_ at 37 °C. After 2 h of anoxia, the cardiomyocytes were subjected to reoxygenation using 95% O_2_ and 5% CO_2_ at 37 °C for 12 h.

### RNA isolation

The total RNA and miRNA-enriched RNA were extracted using TRIzol (Invitrogen, USA) from whole hearts, the mitochondrial fraction of the hearts or cultured primary cardiomyocytes, as per the manufacturer’s recommended protocol.

### Cellular ATP measurements

The cellular ATP content was measured with a firefly luciferase-based ATP determination kit (Molecular Probes). Briefly, cardiomyocytes were washed twice with cold PBS and then lysed with lysis buffer containing phosphatase inhibitors for 30 min at 4 °C. Samples were centrifuged at 13,000 rpm for 15 min at 4 °C, and the supernatant was collected for use in the firefly luciferase-based ATP assay according to the manufacturer’s protocol. The luminescence signal was measured by using a luminometer (TECAN Infinite M200 Pro) at 560-nm absorbance. For each assay, at least 3 replicates were used.

### Mitochondria isolation

The purification of an entire cardiac mitochondrial fraction from the hearts or cultured primary cardiomyocytes was carried out as previously described^51^, using the Mammalian Mitochondria Isolation Kit for Tissue & Cultured Cells (Biovision, Inc) according to the manufacturer’s instructions.

### Measurement of mitochondrial complex activities

Complex I, Complex II, and Complex IV activities of isolated mitochondria were measured using Microplate assay kits (Mitosciences, Inc) according to the manufacturer’s protocol. These enzymes were captured within the wells of the microplate coated by the corresponding complex enzyme antibody, and activities were detected colorimetrically. Complex activities are presented as a percentage activity compared to the heart samples of the control mice.

### ROS measurements

Intracellular ROS levels were measured by incubating cardiomyocytes with dichlorodihydrofluorescein diacetate (H2DCFDA, Molecular Probes), and the DCF fluorescence signal was measured using a fluorophotometer as previously described^51^.

### Transfection of cardiomyocytes with the antagomir, mimics and luciferase reporter plasmids

The chemically modified antagomir complementary to miR-762 was designed to suppress the expression of endogenous miR-762 and the antagomir negative control (anta-NC), which were purchased from GenePharma Co., Ltd. (Shanghai, China). The miR-762 antagomir sequence was 5’-GCUCUGUCCCGGCCCCAGCCCC-3’. The anta-NC sequence was 5’-CAGUACUUUUGUGUAGUACAA-3’. All transient transfections were carried out using lipofectamine 2000 according to the manufacturer’s protocol (Invitrogen, USA).

### Western blot analysis

Western blot analysis was performed as previously described^52^. Briefly, the cardiomyocytes were resuspended in lysis buffer (20 mM Tris pH 7.5, 2 mM EDTA, 3 mM EGTA, 2 mM dithiothreitol, 250 mM sucrose, 0.1 mM phenylmethylsulfonyl fluoride, 1% Triton X-100) with a protease inhibitor cocktail at 4 °C for 1 h. The samples were centrifuged at 13,000 rpm at 4 °C for 30 min. Subsequently, 30 µg of the supernatants was boiled, separated on 10% SDS-polyacrylamide gel and transferred to nitrocellulose membranes. Then, the membranes were incubated at 4 °C overnight with the following diluted primary antibodies. The anti-ND1, anti-ND2, anti-ND3, anti-ND4, anti-ND5, anti-COX1, anti-Cytb, anti-Tom20, and anti-ND6 antibodies were from Abcam. The anti-COX3 and ATP6 antibodies were from Abclonal. The anti-COX2 antibody was purchased from Bimake. The anti-ATP8 and anti-GAPDH antibodies were purchased from Santa Cruz. The anti-ND4L antibody was purchased from Invitrogen. After washing four times with 1× PBST, the membrane was incubated with the appropriate secondary antibody conjugated with horseradish peroxidase (HRP). Antigen–antibody complexes were visualized by enhanced chemiluminescence. The densitometric measurements were analyzed by using Image J software.

### Adenoviral constructions and infection

The adenoviruses harboring ND2 were purchased from Hanbio Biotechnology Co., Ltd (Shanghai, China). All siRNAs, including ND2 and the negative scramble control, were synthesized from GenePharma Co., Ltd (Shanghai, China). The siRNA targeting sequences were as follows: ND2-siRNA: 5’-GGCCATCGTACTCAACTAT-3’; Negative control ND2-sc: 5’-GAAGAATACCCCCAGTTAC-3’. The adenoviruses containing siRNA or their negative scrambled forms were constructed with the pSilencer™ adeno 1.0-CMV System according to the kit’s protocol (Ambion, USA). All viruses were amplified in HEK293 cells.

### qRT-PCR analysis

As described previously^53^, stem-loop qRT-PCR for mature miRNAs was carried out on a CFX96 real-time PCR detection system (Bio-Rad, USA). Total RNA was isolated with TRIzol reagent followed by treatment with RNase-Free DNase I (TaKaRa, Japan). Subsequently, RNA was reverse transcribed using reverse transcriptase (ReverTra Ace, Toyobo). The expression of the indicated miRNAs was normalized by expression of U6 with the 2−ΔΔCt cycle threshold method. The specific U6 primers were Forward: 5’-GCTTCGGCAGCACATATACTAA-3’; Reverse: 5’-AACGCTTCACGAATTTGCGT-3’. qRT-PCR was performed to analyze the expression of ND2. The specific sequences of ND2 primers were forward: 5’-GGCCATCGTACTCAACTATAA-3’; reverse: 5’-GGTAATCAGAAGTGGAATGG-3’. The levels of ND2 mRNA expression were standardized by that of glyceraldehyde-3-phosphate dehydrogenase (GAPDH). The specific sequences of GAPDH primers were forward: 5’-TGTGTCCGTCGTGGATCTGA-3’; reverse: 5’-CCTGCTTCACCACCTTCTTGA-3’. The specificity of the PCR amplification was verified by agarose gel electrophoresis.

### Luciferase reporter gene constructs and luciferase activity assay

We subcloned the CDS of ND2 into the immediate downstream of the stop codon of the luciferase gene of the pGL3 vector (Promega, USA) by PCR amplification. The forward primer was 5’-CGATCAACTGAAGCAGCAACAA-3’, and the reverse primer was 5’-AGTTGAGTAGCGGGTAAATTTG-3’. To generate the pGL3-ND2-CDS-mutant, the introduction of mutations in the putative miR-762 binding site was generated with the QuikChange II XLSite-Directed Mutagenesis Kit (Stratagene, USA) using pGL3-ND2-CDS-wild type as a template. The constructs were sequenced to verify that only the desired mutations had been generated. Luciferase activity was measured using the Dual-Luciferase Reporter Assay System (Promega, USA) according to the company’s recommended protocol.

### Microarray analysis

As described previously^14^, the mitochondrial RNAs were isolated using TRIzol (Invitrogen, USA) from the cardiomyocytes treated with A/R. Subsequently, an Affymetrix miRNA microarray was used to analyze the expression profiles of miRNAs with the Affymetrix® GeneChip® miRNA Array 3.0 according to the manufacturer’s recommended protocol (CapitalBio Corp., Beijing, China).

### Fluorescence in situ hybridization

For mitochondrial staining, primary neonatal cardiomyocytes plated for 2 days were incubated with 0.02 µM MitoTracker Red CMXRos for 30 min in live cell staining with mitochondrial membrane potential dye (Invitrogen, GrandIsland, USA) according to the manufacturer’s protocol^14^. Then, cardiomyocytes were fixed in acid methanol followed by drying overnight. MiR-762 was detected by fluorescence in situ hybridization with labeled nucleic acid (LNA) probes as previously described^14,54^. Briefly, the coverslips were fixed in acid methanol (60% methanol and 10% acetic acid) and dried overnight. Subsequently, cardiomyocytes were hybridized in situ for 1 hat 56 °C with LNA^TM^ microRNA probe double DIG-labeled miR-762 (Exiqon, QIAGEN, USA) followed by washing with 1× SSC buffer (Invitrogen, USA) at 56 °C. After a DIG buffer wash (Roche Diagnostics, Mannheim, Germany), 3% hydrogen peroxide solution was used to quench endogenous peroxidase activity followed by a TNB block (Perkin Elmer, Waltham, MA). The cardiomyocyteswere incubated with Anti-DIG POD (Roche) for 30 min. Finally, TSA Plus Fluorescein Amplification Reagent was used to incubate the cardiomyocytes followed by a series of washes with TNT buffer, and we removed the chambers and coverslipped the slides with Vectashield using DAPI (Vector Labs, Burlingame, CA). In contrast, control slides were hybridized using no probe or incubated with no TSA Plus Fluorescein Amplification reagent. The fluorescence was imaged using a laser scanning confocal microscope (Zesis LSM510 META).

### Echocardiographic assessment

The mice were subjected to transthoracic echocardiographic analysis one week after the sham or I/R surgery as previously described^55^. Echocardiographic parameters, including systolic left ventricular internal diameters (LVIDs) and diastolic left ventricular internal diameters (LVIDd), were measured by echocardiography, and fractional shortening (FS) of left ventricular diameter was calculated as follows: FS (%) = [(LVIDd –LVIDs)/LVIDd] × 100. Subsequently, the mice were subjected to anesthetization, and the hearts were collected, weighed and used for post-mortem histological examination.

### Oxygen consumption rate

Cardiomyocytes were seeded at a density of 2 × 10^5^ cells/well into Seahorse Bioscience XF microplates and grown in DMEM. After plating for 24 h, the cardiomyocytes were transfected with anta-NC or anta-762 or no transfection. After 36 h, the cardiomyocytes were subjected to A/R treatment or no treatment condition. Then, cardiomyocytes were incubated with assay media (XF base medium supplemented with 10 mM glucose, 2 mM L-glutamine, and 2 mM pyruvate, pH 7.4) in a CO_2_-free incubator at 37 °C for 1 h before loading the plate in the XFp analyzer. Oxygen consumption was measured using a Seahorse XFp Analyzer (Seahorse Biosciences; North Billerica, MA, USA) with the XF Cell Mito Stress Test, oligomycin (1 μmol/L), FCCP (2 μmol/L), and rotenone (0.5 μmol/L) plus antimycin A (0.5 μmol/L) were sequentially added to each well at specified time points. The calculations for basal respiration, proton leak and ATP linked were determined as described in the Seahorse Operator’s Manual. All experiments were performed at least three times.

### Animal experiment

Male adult C57BL/6 mice (10–12-week-old) were purchased from the Institute of Laboratory, Animal Science of Chinese Academy of Medical Sciences (Beijing, China). For the I/R injury model, mice were subjected to 1 h ischemia followed by reperfusion for 3 h or 1 week as previously described^50^. Sham-operated mice underwent the same surgery with the exception of the snare, which was left untied. For the delivery of the antagomir miR-762 (anta-762) or antagomir NC (anta-NC), the mice were subjected to treatment for three consecutive days with intravenous injections of antagomir miR-762 or its control at a dose of 40 mg/kg (0.2 ml per injection). For the intracoronary delivery of adenoviruses, the mice were subjected to anesthetization and ventilation with a small-animal ventilator (HX-300S, TME, China). A small left anterior thoracotomy was performed on the left side of the chest, followed by removal of the pericardial sac. We injected adenoviruses harboring ND2 (2 × 10^11^moi) with a catheter from the apex of the left ventricle into the aortic root and at the same time cross-clamped the aorta and the pulmonary arteries. The clamp was sustained for 20 s with heart pumping against a closed system. Subsequently, the chest wall was sewn up, and the mice were transferred back to clean warm cages for recovery. Then, the mice were subjected to I/R surgery five days after the injection of adenoviruses.

To calculate infarct sizes, a 2% Evans Blue solution (Sigma-Aldrich, USA) was injected into the jugular vein in the heart to demarcate the nonischemic myocardium. The heart was rapidly excised, and the slices were stained with 1.0% 2,3,5-triphenyltetrazolium chloride (Sigma-Aldrich, USA) at 37 °C for 15 min, demarcating the viable and nonviable myocardium within the area at risk (AAR). Subsequently, ice-cold sterile saline was added to stop the staining procedure, and the slices were fixed in 10% neutral buffered formaldehyde. We calculated the areas of infarction (INF) and nonischemic left ventricle (LV) by computer-assisted planimetry (NIH Image 1.57) as previously described^50^. All animal experiments were approved by the QingDao University Animal Care Committee.

### Apoptosis assays

Apoptosis was measured by terminal deoxyribonucleotidyl transferase-mediated TdT-mediated dUTP nick end labeling (TUNEL) with a kit from Roche according to the company’s recommended protocol. The procedures were performed according to the manufacturer’s protocol (Roche, Germany). The percentage of apoptotic cells was calculated as the ratio of TUNEL-positive cells to the total nuclei. The experiments were repeated at least three times.

### RNA immunoprecipitation

As described previously^56^, RNA immunoprecipitation was carried out using an Ago2-specific antibody, and a normal IgG antibody served as a negative control. Briefly, cardiomyocytes were harvested and lysed in 150 mM KCl, 25 mM Tris-HCl, pH 7.4, 5 mM EDTA, 0.5% Triton X-100, and 5 mM DTT containing Ribolock (Fermentas MBI, Pittsburgh, PA, USA) and proteinase inhibitor cocktail (Roche Applied Science). The samples were incubated with the appropriate Ago2-specific antibody or normal IgG antibody for 1 h at 4 °C. Subsequently, Protein A/G agarose was added to the above samples. After rotation for 4 h at 4 °C, the beads were washed six times using lysis buffer. Finally, half of the beads were used to elute the bound protein with 100 mM glycine, and the remaining beads were used to isolate the RNA using TRIzol reagent (Invitrogen, USA).

### Statistical analysis

The results are presented as the mean ± SEM. Statistical analysis for comparison of two groups was performed using two-tailed unpaired Student’s *t*-test. One-way analysis of variance (ANOVA) followed by Tukey’s post hoc test is used to evaluate differences of more than two groups. Adjusted two-sided *P*-values were calculated and *P* < 0.05 was considered statistically significant.

## Supplementary information

supplementary Figure legends.

Supplementary Table. Microarray analysis of miRNAs induced by A/R in mitochondria.

supplementary Figure 1.

supplementary Figure 2.

supplementary Figure 3.
