# Supplementary material for: Mitochondrial miR-762 regulates apoptosis and myocardial infarction by impairing ND2
Source: Cell Death Dis. 2019 Jun 24;10(7):500. doi: 10.1038/s41419-019-1734-7 (PMC6591419; doi:10.1038/s41419-019-1734-7)
Supplement: Supplementary file 1 — supplementary Figure legends. [file 41419_2019_1734_MOESM1_ESM.docx]

**Figure S1. Role of miR-762 in the mitochondrial respiration rate and energy production.**

Oxygen consumption rate (OCR) measurements for (**a**) basal respiration, (**b**) ATP linked, (**c**) proton leak using a Seahorse XFp analyzer following sequential addition of oligomycin (1 μmol/L), FCCP (2 μmol/L), and rotenone (0.5 μmol/L) plus antimycin A (0.5 μmol/L). Results in (**a**) are plotted relative to normal condition set to 100. Data in (**b**) and (**c**) are expressed as percent of basal respiration. n=3. **P*<0.05. Data are shown as the mean±SEM and analysed with one-way ANOVA followed by Tukey-Kramer post hoc test.

**Figure S2. The effect of miR-762 on the mRNA and protein levels of mitochondrial protein coding genes.**

**a** Enforced of miR-762 had no distinct effect on the mRNA levels of mitochondrial protein coding genes including ND3, ND4, ND4L, ND5, COX2, COX3 and ATP6. Cardiomyocytes were transfected with anta-762 or anta negative control (anta-NC). The expression of mitochondrial genes was detected by qRT-PCR (n=3). Data are shown as the mean±SEM and analysed with one-way ANOVA followed by Tukey-Kramer post hoc test. **b** Enforced of miR-762 had no distinct effect on the levels of mitochondrial DNA-coded proteins including ND3, ND4, ND4L, ND5, COX2, COX3 and ATP6. Cardiomyocytes were transfected with anta-762 or anta-NC. The levels of proteins were analyzed by western blot analysis.

**Figure S3. Enforced expression of miR-762 attenuates the expression of ND2.**

**a** Enforced expression of miR-762 had no distinct effect on the expression of ND2-CDS-mut. Cardiomyocytes were transfected with ND2-CDS-mut with mimics as described in the Methods section. The expression of ND2 was detected by western blot analysis. **b** Enforced expression of miR-762 dramatically attenuated the expression of ND2-CDS-wt. Cardiomyocytes were transfected with ND2-CDS-wt with mimics as described in the Methods section. The expression of ND2 was detected by western blot analysis. **c** Enforced expression of miR-762 dramatically reduced intracellular ATP levels. Intracellular ATP levels were determined after transfection with ND2-CDS-wt, ND2-CDS-mut or mimics. ATP levels were determined as described in the methods (n=3). **P*<0.05. Data are shown as the mean±SEM and analysed with one-way ANOVA followed by Tukey-Kramer post hoc test.
