# Supplementary material for: Mitochondrial miR-762 regulates apoptosis and myocardial infarction by impairing ND2
Source: Cell Death Dis. 2019 Jun 24;10(7):500. doi: 10.1038/s41419-019-1734-7 (PMC6591419; doi:10.1038/s41419-019-1734-7)
Supplement: Supplementary file 2 — Supplementary Table. Microarray analysis of miRNAs induced by A/R in mitochondria. [file 41419_2019_1734_MOESM2_ESM.doc]

**Supplementary Table. Microarray analysis of miRNAs induced by A/R in mitochondria.**

| **ProbeSetID** | **Average intensities** | | **Fold change**  **(A/R/control)** |
| --- | --- | --- | --- |
|  | **A/R** | **Control** |  |
| **Upregulated genes** | | | |
| mmu-miR-744_st | 459.9408 | 139.3544 | 3.3005 |
| mmu-miR-92a-star_st | 305.6729 | 111.949 | 2.7305 |
| mmu-miR-1892_st | 965.308 | 385.3519 | 2.505 |
| mmu-miR-150-star_st | 95.92898 | 38.54163 | 2.489 |
| mmu-miR-669a_st | 105.6428 | 43.76235 | 2.414 |
| mmu-miR-762_st | 664.9619 | 312.629 | 2.127 |
| mmu-miR-296-3p_st | 77.42258 | 36.67845 | 2.1108 |
| mmu-miR-711_st | 490.0605 | 237.8449 | 2.0604 |
| mmu-miR-450a-3p_st | 118.0761 | 58.40192 | 2.0218 |
| **Downregulated genes** | | | |
| mmu-miR-362-5p_st | 34.56499 | 69.37975 | 0.4982 |
| mmu-miR-532-5p_st | 92.5873 | 186.40487 | 0.4967 |
| mmu-miR-31-star_st | 16.33563 | 33.26335 | 0.4911 |
| mmu-miR-139-5p_st | 86.57793 | 176.7865 | 0.4897 |
| mmu-miR-330-star_st | 17.30566 | 36.09991 | 0.4794 |
| mmu-miR-379_st | 51.89718 | 122.7622 | 0.4227 |
