# Supplementary figures and images for: Mitochondrial miR-762 regulates apoptosis and myocardial infarction by impairing ND2
Source: Cell Death Dis. 2019 Jun 24;10(7):500. doi: 10.1038/s41419-019-1734-7 (PMC6591419; doi:10.1038/s41419-019-1734-7)

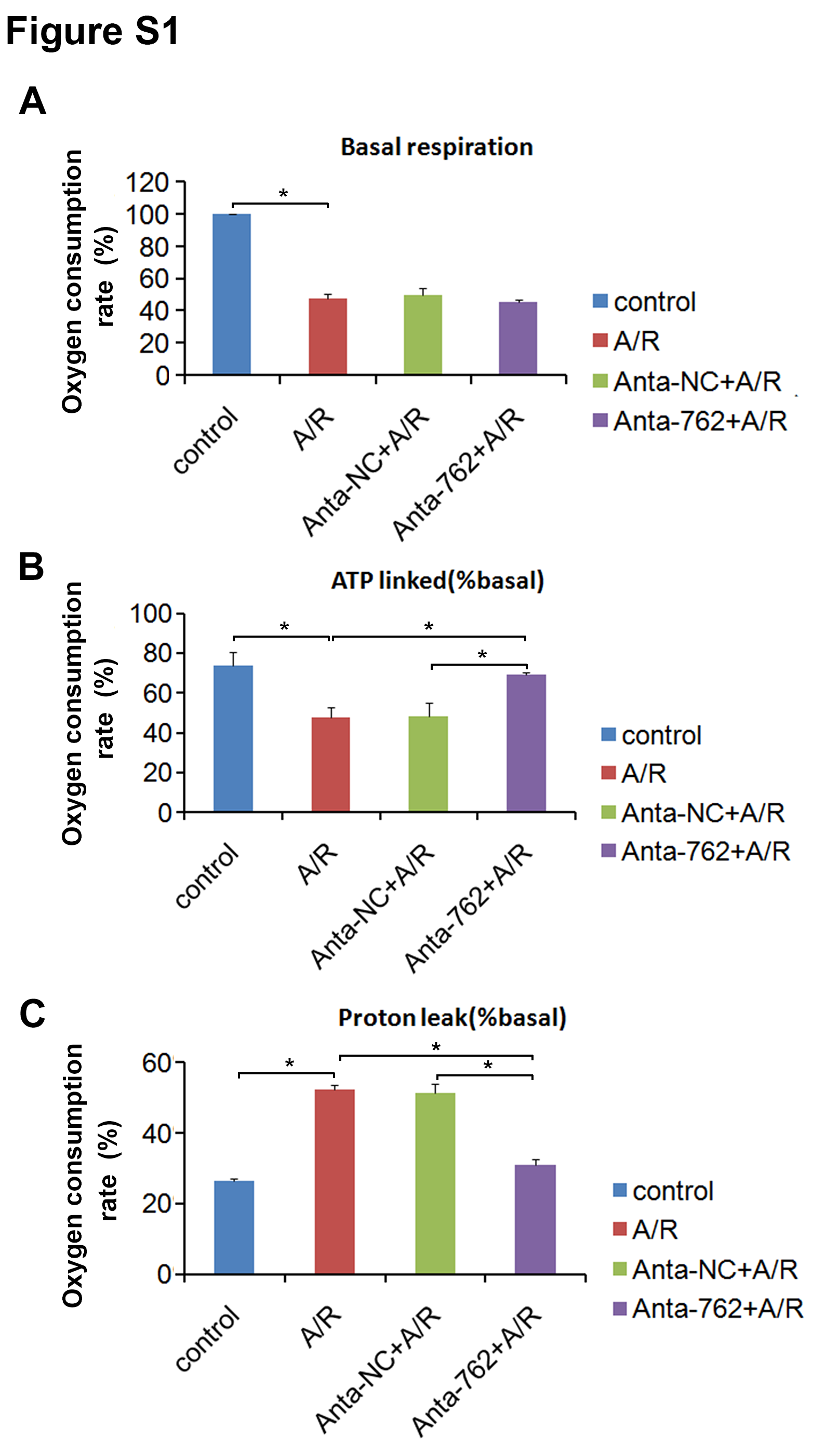

Supplement: Supplementary file 3 — supplementary Figure 1. [file 41419_2019_1734_MOESM3_ESM.tif]

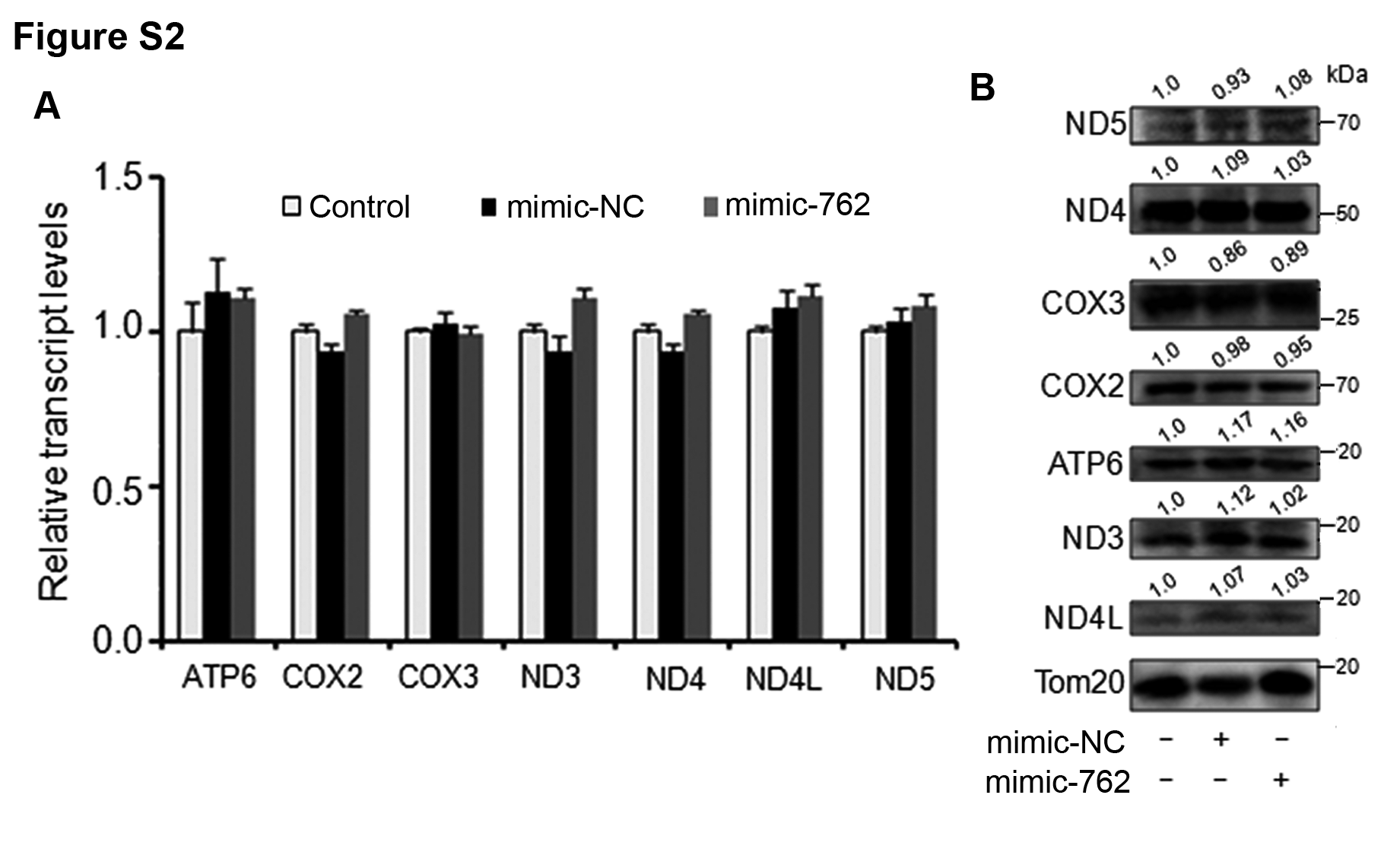

Supplement: Supplementary file 4 — supplementary Figure 2. [file 41419_2019_1734_MOESM4_ESM.tif]

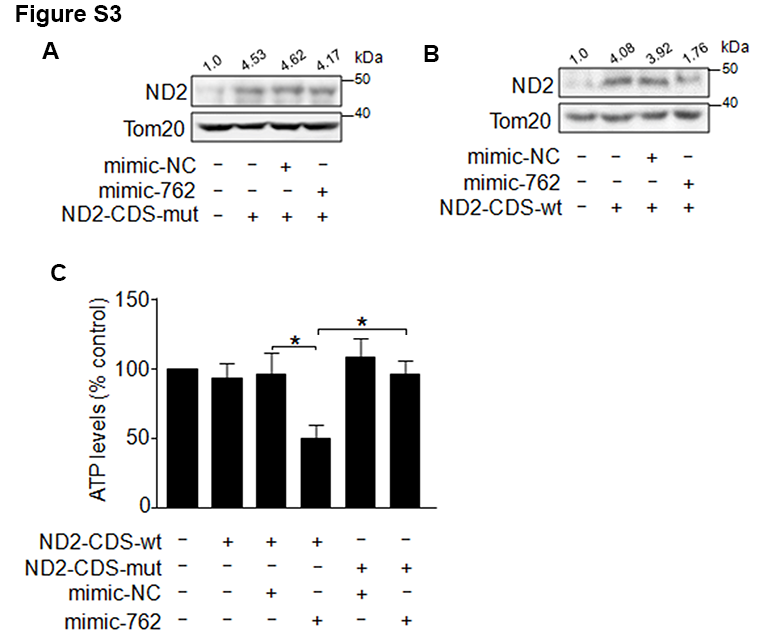

Supplement: Supplementary file 5 — supplementary Figure 3. [file 41419_2019_1734_MOESM5_ESM.tif]
